# AMPK-independent inhibition of human macrophage ER stress response by AICAR

**DOI:** 10.1038/srep32111

**Published:** 2016-08-26

**Authors:** Marcel Boß, Yvette Newbatt, Sahil Gupta, Ian Collins, Bernhard Brüne, Dmitry Namgaladze

**Affiliations:** 1 Institute of Biochemistry I, Goethe-University Frankfurt, Theodor-Stern-Kai 7, 60596 Frankfurt, Germany; 2 Division of Cancer Therapeutics, Institute of Cancer Research, Sutton, Surrey SM2 5NG, UK; 3 Project Group Translational Medicine and Pharmacology TMP, Fraunhofer Institute for Molecular Biology and Applied Ecology IME, Theodor-Stern-Kai 7, 60596 Frankfurt, Germany

## Abstract

Obesity-associated insulin resistance is driven by inflammatory processes in response to metabolic overload. Obesity-associated inflammation can be recapitulated in cell culture by exposing macrophages to saturated fatty acids (SFA), and endoplasmic reticulum (ER) stress responses essentially contribute to pro-inflammatory signalling. AMP-activated protein kinase (AMPK) is a central metabolic regulator with established anti-inflammatory actions. Whether pharmacological AMPK activation suppresses SFA-induced inflammation in a human system is unclear. In a setting of hypoxia-potentiated inflammation induced by SFA palmitate, we found that the AMP-mimetic AMPK activator 5-aminoimidazole-4-carboxamide-1-β-D-ribofuranoside (AICAR) potently suppressed upregulation of ER stress marker mRNAs and pro-inflammatory cytokines. Furthermore, AICAR inhibited macrophage ER stress responses triggered by ER-stressors thapsigargin or tunicamycin. Surprisingly, AICAR acted independent of AMPK or AICAR conversion to 5-aminoimidazole-4-carboxamide-1-β-D-ribofuranosyl monophosphate (ZMP) while requiring intracellular uptake via the equilibrative nucleoside transporter (ENT) ENT1 or the concentrative nucleoside transporter (CNT) CNT3. AICAR did not affect the initiation of the ER stress response, but inhibited the expression of major ER stress transcriptional effectors. Furthermore, AICAR inhibited autophosphorylation of the ER stress sensor inositol-requiring enzyme 1α (IRE1α), while activating its endoribonuclease activity *in vitro*. Our results suggest that AMPK-independent inhibition of ER stress responses contributes to anti-inflammatory and anti-diabetic effects of AICAR.

The importance of metabolism in regulating immunity and inflammation is now widely recognized^1^,^2^. AMP-activated protein kinase (AMPK) is the central hub of metabolic regulation, rewiring cellular metabolic fluxes in response to energy loss to decrease anabolic and increase catabolic processes^3^. In innate immune cells, AMPK suppresses inflammatory signalling^4^ and promotes anti-inflammatory macrophage polarization^5^. The anti-inflammatory potential of AMPK is particularly interesting because of its potential application for anti-diabetic therapies. Current concepts on the development of insulin resistance and type 2 diabetes consider chronic inflammatory response caused by obesity-driven adipose tissue expansion to be a major contributing factor to disease progression. Macrophages recruited to the expanding adipose tissue undergo pro-inflammatory polarization^6^,^7^, which can be mimicked *in vitro* by macrophages exposure to saturated fatty acids (SFA), such as palmitate^7^,^8^,^9^. Mechanistically, SFA-induced inflammation was explained by activation of toll-like receptors^10^,^11^ as well as induction of intracellular stress signalling cascades, in particular, c-Jun N-terminal kinase (JNK)^12^ and endoplasmic reticulum (ER) stress response^13^. ER stress responses, mediated by the activation of three major upstream effectors, i.e. protein kinase R-like endoplasmic reticulum kinase (PERK), endoribonuclease inositol-requiring enzyme 1 (IRE1), and the activating transcription factor 6 (ATF6), can be triggered by SFAs through increasing the degree of saturation of ER membrane phospholipids^14^, provoking direct activation of PERK and IRE1. IRE1, in turn, activates JNK via IRE1 association with the adaptor protein tumour necrosis factor receptor associated factor 2 (TRAF2)^15^. SFA-induced ER stress, together with other mechanisms, such as increased Src kinase association with lipid rafts^16^, connect fatty acid metabolism to inflammatory signalling. On the other hand, increasing fatty acid mitochondrial oxidation, by diverting the fatty acid flux towards catabolism, attenuates ER stress and inflammation in palmitate-treated macrophages^17^. AMPK is a well-known activator of fatty acid oxidation (FAO) and indeed its absence in macrophages increased palmitate-induced inflammatory responses, partly through diminished FAO^18^. An alternative mechanism how AMPK attenuates inflammation in palmitate-treated macrophages suggestively involves activation of the protein deacetylase (Sirt1)^19^.

Considering the therapeutic potential of AMPK during metabolic and inflammatory disorders, it is critical to understand mechanistic aspects and possible off-target effects of clinically employed AMPK activators. AMPK activators can be divided in three classes: i) molecules converted to AMP mimetics, such as 5-aminoimidazole-4-carboxamide-1-β-D-ribofuranoside (AICAR), ii) allosteric activators such as A769662 or iii) drugs inhibiting mitochondrial ATP production and elevating AMP, such as the widely used anti-diabetic drug metformin^20^. Most of AMPK activators also show biological effects unrelated to AMPK activation. Importantly, AMPK-independent inhibition of inflammation was reported for metformin^21^ and for AICAR^22^,^23^. However, the exact mechanism of how AICAR inhibits inflammation is still unresolved.

Here we report that AICAR profoundly inhibits SFA-induced ER stress and inflammatory responses to palmitate in human macrophages. Furthermore, AICAR turned out as a general inhibitor of the ER stress response, which occurs independently of the conversion of AICAR to 5-aminoimidazole-4-carboxamide-1-β-D-ribofuranosyl monophosphate (ZMP) and hence, AMPK activation. Mechanistically, AICAR inhibited mRNA and protein induction of the major transcriptional effectors of the ER stress response, without interfering with initiation of ER stress.

## Results

### AICAR inhibits hypoxia-enhanced palmitate-induced inflammation in human macrophages

**Figure 1:**
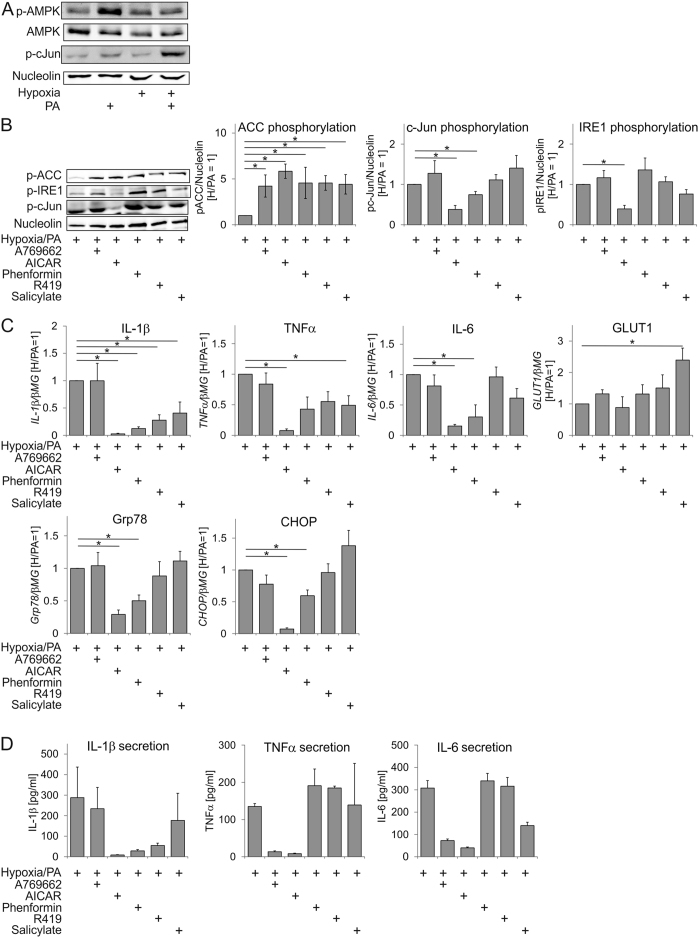
AICAR inhibits hypoxia/palmitate-induced ER stress and inflammation in hypoxic macrophages. (**A**,**B**) Western analysis of macrophages exposed to palmitate (PA) in the presence or absence of hypoxia and AMPK activators for 24 h. (**C**) mRNA expression of indicated genes in hypoxic macrophages treated for 24 h with palmitate and AMPK activators. (**D**) Cytokine secretion in hypoxic macrophages treated for 24 h with palmitate and AMPK activators. *p < 0.05. Data represent mean values ± SE of at least three independent experiments. Western Blot images are cropped scans either of the same or of the duplicate membranes probed with different antibodies.

AMPK activation is considered to be anti-inflammatory^1^, but the ability of pharmacologic AMPK activators to block inflammatory responses is still unclear. We used an experimental system of hypoxia-enhanced palmitate-induced inflammation in primary human macrophages^8^ to test distinct classes of AMPK activators for their effect on palmitate-induced ER stress and inflammatory markers. This experimental system reflects a pro-inflammatory, hypoxic milieu of hypertrophic adipose tissue in obesity. Analysing phosphorylation of AMPK at T172 as a marker of AMPK activation, we noticed that palmitate moderately activated AMPK, while hypoxia reduced this activity (Fig. 1A), suggesting that AMPK is not activated under palmitate/hypoxic exposure. As observed previously^8^, c-Jun phosphorylation, a readout of an inflammatory response, increased under hypoxia/palmitate (Fig. 1A). We then chose drugs activating AMPK allosterically (A769662, salicylate), changing the adenylate energy charge (phenformin, R419), or mimicking AMP (AICAR)^24^,^25^ at concentrations causing similar AMPK activation as followed by phosphorylation of the AMPK substrate acetyl-CoA carboxylase (ACC) (Fig. 1B). In parallel, we analysed the markers of ER stress, i.e. phospho-IRE1, and inflammation (phospho-cJun). Of all AMPK activators only AICAR consistently inhibited palmitate-induced IRE1 and cJun phosphorylation (Fig. 1B). mRNA expression of ER stress-responsive genes *Grp78* and *CHOP* as well as inflammatory cytokines *IL-1β*, *TNFα*, and *IL-6* confirmed that AICAR was the most potent inhibitor (Fig. 1C). Among other AMPK activators only phenformin inhibited ER stress responses and cytokines, whereas R419 and salicylate reduced only *IL1β* and *IL6* mRNA expression. A769662 was without any effect at all. None of the AMPK activators inhibited the expression of hypoxia-sensitive *GLUT1* mRNA, suggesting that signalling through hypoxia-inducible factor (HIF) remains intact. Analysis of IL1β, TNFα and IL-6 protein secretion after hypoxia/palmitate treatment revealed divergent effects of AMPK activators on different cytokines (Fig. 1D). Remarkably, only AICAR consistently inhibited the secretion of all three cytokines. Our data indicate that AMPK activators suppress SFA-triggered inflammatory responses to a variable degree, suggesting that AICAR may block palmitate-induced ER stress and inflammation through mechanisms unrelated to AMPK activation.

We went on to investigate mechanisms how AICAR inhibits SFA-induced inflammatory responses. Although previous studies suggested the involvement of Sirt1 and FAO in attenuating SFA-induced inflammation by AMPK^18^,^19^, the AICAR effect in our study was neither reversed by the Sirt1 inhibitor Ex527, nor by the FAO blocker etomoxir (Supplementary Figure S1A). Active AMPK also interferes with mechanistic target of rapamycin complex 1 (mTORC1), which may attenuate inflammatory cell responses^1^. However, treatment of macrophages with the mTORC1 inhibitor rapamycin did not influence the expression of inflammatory and ER stress markers after hypoxia/palmitate (Supplementary Figure S1A). Furthermore, AICAR did not affect triglyceride levels in palmitate-treated hypoxic macrophages (Supplementary Figure S1B), suggesting that changes in fatty acid metabolism are unlikely to explain AICAR effects.

### AICAR inhibits ER stress responses in macrophages in AMPK-independent manner

**Figure 2:**
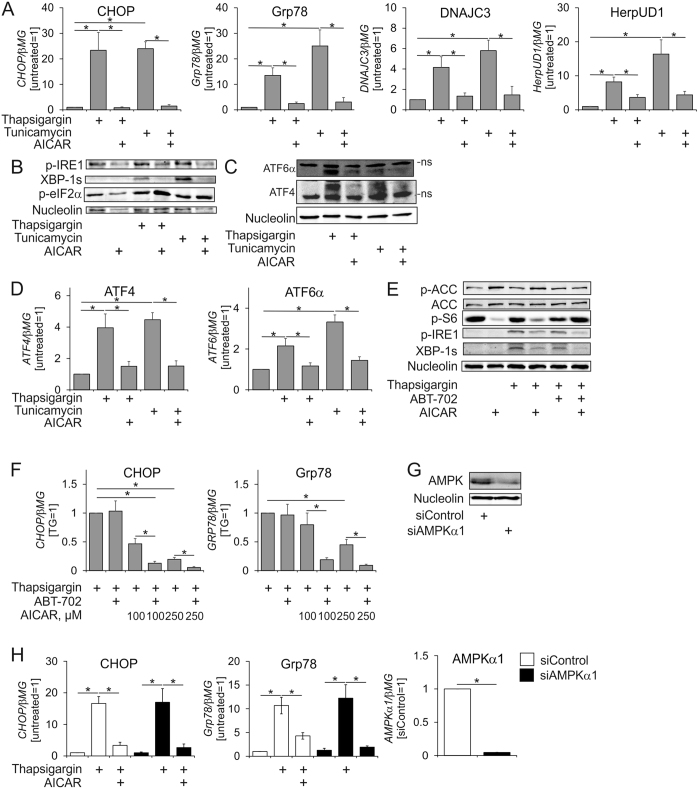
AICAR inhibits ER stress responses. (**A**) mRNA expression of ER stress markers, (**B**,**C**) Western analysis of cell lysates (**B**) or nuclear extracts (**C**).(**D**) mRNA expression of ATF4 and ATF6α in macrophages pre-exposed for 1 h to AICAR and treated with thapsigargin or tunicamycin for 6 h. (**E**) Western analysis of macrophages pre-exposed for 1 h to 0.5 mM AICAR in the absence or presence of ABT-702 and treated with thapsigargin for 6 h. (**F**) mRNA expression of CHOP and Grp78 in macrophages pre-exposed for 1 h to indicated concentrations of AICAR in the absence or presence of ABT-702 and treated with thapsigargin or tunicamycin for 6 h. (**G**) Protein expression of AMPK 72 h post-transfection with control siRNA or AMPKα1 siRNA. (**H**) mRNA expression of CHOP, Grp78 and AMPKα1 in macrophages transfected with control siRNA or AMPKα1 siRNA for 72 h prior to treatments with thapsigargin for 6 h with or without 1 h AICAR pre-exposure. *p < 0.05. Data represent mean values ± SE of at least three independent experiments. ns, non-specific band. Western Blot images are cropped scans either of the same or of the duplicate membranes probed with different antibodies.

With no indication towards the previously described anti-inflammatory mechanisms of AMPK in our system, we followed the observation that only AICAR suppressed both, IRE1 phosphorylation and ER stress markers in palmitate-treated macrophages. Thus, we questioned whether AICAR acts as a general inhibitor of ER stress responses in macrophages. Therefore, we used the prototype ER stress inducers thapsigargin and tunicamycin in cells pre-treated with AICAR. AICAR largely attenuated induction of ER stress mRNA markers by thapsigargin and tunicamycin (Fig. 2A).

ER stress responses are executed by three major branches initiated by the ER stress sensors PERK, IRE1, and ATF6. To evaluate whether AICAR inhibits all three branches we examined PERK activation by following phosphorylation of the eukaryotic initiation factor 2α (eIF2α), a known PERK substrate. IRE1 activation was assessed by analysing pIRE1 and protein amounts of spliced X-box binding protein 1 (XBP1) as a readout of IRE1 activity. In addition, we assessed the ATF6 branch by following accumulation of cleaved ATF6 in nuclear lysates. AICAR blocked thapsigargin and tunicamycin-induced IRE1 phosphorylation, accumulation of spliced XBP1 (Fig. 2B), but did not affect eIF2α phosphorylation. However, AICAR prevented the nuclear accumulation of activating transcription factor 4 (ATF4), a target and a major mediator of the PERK ER stress response pathway (Fig. 2C). We also noticed that AICAR blocked ATF4 mRNA induction by ER stressors (Fig. 2D), providing a possible explanation for the lack of ATF4 protein accumulation. Furthermore, nuclear levels of cleaved ATF6α protein as well as ATF6α mRNA expression after ER stress were also diminished in AICAR-treated macrophages (Fig. 2C,D). Thus, all three branches of the ER stress response are inhibited by AICAR.

To further investigate mechanisms how AICAR inhibits the ER stress response we first questioned whether AICAR demands AMPK activity or relies on AICAR conversion to ZMP, a step critical for AICAR to become an AMPK activator. AICAR phosphorylation to ZMP is catalysed by adenosine kinase. Addition of a potent adenosine kinase inhibitor ABT-702 prevented AICAR-induced AMPK activation as seen by the lack of ACC phosphorylation in AICAR-treated cells (Fig. 2E). Furthermore, AICAR-triggered inhibition of mTOR, analysed by following the phosphorylation of ribosomal S6 protein, was abolished by ABT-702 (Fig. 2E). However, ABT-702 did not prevent the impact of AICAR on IRE1 phosphorylation or XBP1 splicing (Fig. 2E). In the presence of ABT-702, the inhibitory effect of AICAR on IRE1 phosphorylation and XBP1 splicing even appeared to be enhanced. Indeed, ABT-702 potentiated the ability of low AICAR concentrations to inhibit mRNA expression of ER stress markers (Fig. 2F). Collectively, these data suggested that AICAR acts upstream of its conversion to ZMP and independently of AMPK. Supporting these data, a knockdown of AMPK α1 catalytic subunit (Fig. 2G) did not prevent inhibition of CHOP and Grp78 gene expression by AICAR (Fig. 2H). Dissociation of AICAR inhibition of ER stress and inflammatory response from AMPK activation was also observed in concentration-dependence experiments in palmitate-treated hypoxic macrophages (Supplementary Figure S2).

### Nucleoside transporters involved into AICAR trafficking into macrophages

**Figure 3:**
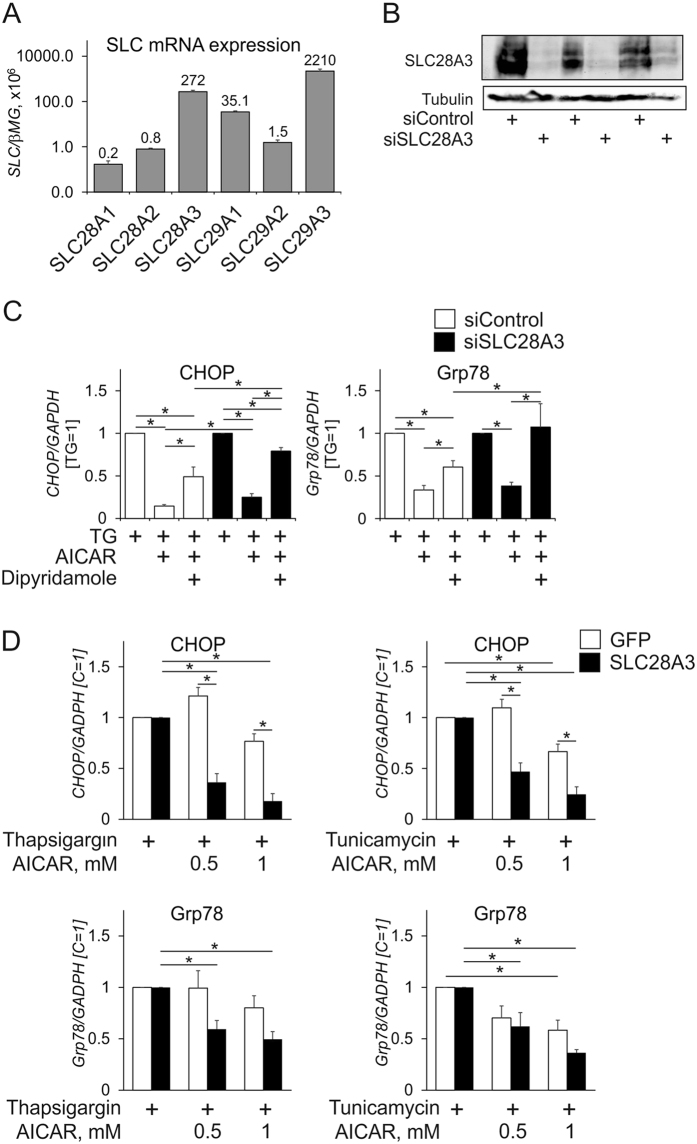
SLC29A1 and SLC28A3 facilitate the uptake of AICAR into macrophages. (**A**) mRNA expression of SLC28 and SLC29 family members in macrophages. (**B**) Protein expression of SLC28A3 72 h post-transfection with control siRNA or SLC28A3 siRNA. (**C**) mRNA expression of CHOP and Grp78 in macrophages transfected with control siRNA or SLC28A3 siRNA for 72 h prior to treatments with thapsigargin for 6 h with or without 1-h AICAR pre-exposure in the absence or presence of dipyridamole. (**D**) mRNA expression of CHOP and Grp78 in HEK293T cells transfected with GFP or SLC28A3 expressing plasmids and treated with thapsigargin or tunicamycin for 6 h with or without 1 h pre-exposure to indicated concentrations of AICAR. *p < 0.05. Data represent mean values ± SE of at least three independent experiments. Western Blot images are cropped scans of the same membrane probed with different antibodies.

Although we observed pronounced effects of AICAR on ER stress responses in macrophages, these effects could not be reproduced in different cell lines, such as THP-1 monocytic cells or HEK293T cells (data not shown). We reasoned that differences in intracellular AICAR accumulation may explain cell type-specific effects of AICAR. Previous studies suggested that most of the AICAR trafficking into cells is mediated by dipyridamole-sensitive adenosine transporters of equilibrative nucleoside transporter (ENT) family (SLC29A1-4), but also concentrative nucleoside transporter (CNT) CNT3 (SLC28A3) can transport AICAR into cells^26^,^27^. Assessing mRNA levels of individual SLC28 and SLC29 family members in human primary macrophages revealed abundant expression of SLC29A3 and SCL28A3 mRNAs (Fig. 3A; note the logarithmic scale of the graph), moderate amounts of SLC29A1 mRNA, whereas SLC29A2, SLC28A1, and SLC28A2 were marginally expressed. To question the roles of individual nucleoside transporters in AICAR - ER stress responses we inhibited SLC29A1/A2 with dipyridamole alone or in combination with silencing SLC28A3 using RNA interference. This was followed by the analysis of mRNA expression of ER stress markers CHOP and Grp78 in response to thapsigargin. Silencing efficiency was verified by western analysis of SLC28A3 protein (Fig. 3B). Combining dipyridamole treatment with SLC28A3 silencing abolished the ability of AICAR to inhibit ER stress responses, whereas single treatments were only partly effective (Fig. 3C). Furthermore, overexpression of SLC28A3 in HEK293T cells allowed inhibition of ER stress responses to thapsigargin or tunicamycin. This became evidenced by an attenuated CHOP and Grp78 expression, whereas AICAR showed limited efficacy in the absence of overexpressed SLC28A3 (Fig. 3D). In contrast, silencing of SLC29A3 did not influence the inhibition of ER stress marker mRNA expression by AICAR (data not shown). These data suggest that entry of AICAR into cells through SLC29A1 and SLC28A3 transporters is necessary to interfere with ER stress in macrophages.

### Effects of AICAR on IRE1α activity *in vitro*

**Figure 4:**
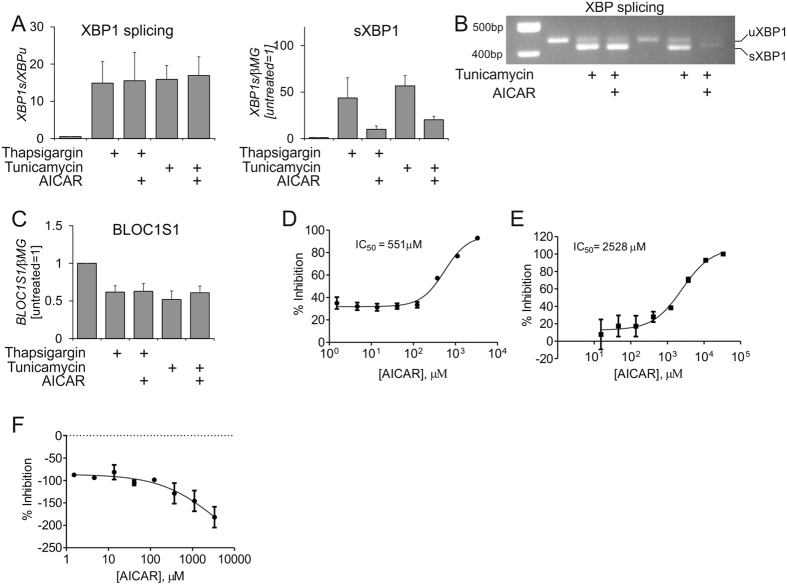
AICAR inhibits IRE1α kinase, but not its splicing activity. (**A**) Q-PCR analysis of XBP1 splicing in macrophages pre-exposed for 1 h to AICAR and treated with thapsigargin or tunicamycin for 6 h. (**B**) RT-PCR analysis of XBP1 splicing in cells treated with AICAR and tunicamycin. The spliced (sXBP1) and unspliced (uXBP1) forms of XBP1 are indicated. (**C**) mRNA expression of BLOC1S1 in macrophages pre-exposed for 1 h to AICAR and treated with thapsigargin or tunicamycin for 6 h. (**D**) Analysis of AICAR binding to recombinant IRE1α. (**E**,**F**) Analysis of recombinant IRE1α kinase (**E**) and endoribonuclease (**F**) activity in the presence of indicated AICAR concentrations. *p < 0.05. Data represent mean values ± SE of at least three independent experiments.

With the information that AICAR prevented accumulation of phosphorylated IRE1 and spliced XBP1 proteins in ER-stressed macrophages we investigated the effects of AICAR on the IRE1-XBP1 branch of the UPR in more detail. Contrary to our expectations, analysis of XBP1 splicing using primers specific to spliced and unspliced XBP1 mRNA revealed that the ER stress-induced splicing activity of IRE1 was not affected by AICAR (Fig. 4A,B). However, we observed lower mRNA levels of spliced XBP1, suggesting that XBP1 mRNA expression was attenuated by AICAR. IRE1 endoribonuclease activity also participates in a process of regulated IRE1-dependent decay (RIDD) of select mRNAs. To evaluate the influence of AICAR on RIDD, we analysed the mRNA expression of a well-characterized RIDD target mRNA BLOC1S1. Indeed, ER stress reduced BLOC1S1 mRNA expression in macrophages, which was unaffected by AICAR (Fig. 4C). Finally, we analysed the interaction of AICAR with recombinant IRE1α *in vitro*. AICAR bound to the ATP-binding site of IRE1α as evidenced by the Lanthascreen assay (Fig. 4D), and inhibited the IRE1α autophosphorylation in a DELFIA assay (Fig. 4E). However, when assessing endoribonuclease activity of IRE1α, AICAR appeared to activate IRE1α (Fig. 4F), which is consistent with the properties of reported type I kinase inhibitors of IRE1α^28^,^29^. Thus, AICAR binds IRE1α and inhibits IRE1α kinase, but not its endoribonuclease activity *in vitro*. This is in agreement with the observations in cells.

Collectively, our observations indicate that AICAR, while not affecting initiation steps of the UPR, prevents accumulation of transcriptional effectors of the UPR, i.e., ATF4, sXBP1, and ATF6α both at mRNA and protein level. Remarkably, this effect is independent of AICAR to be converted to ZMP and to activate AMPK.

## Discussion

The novel information of this study is the ability of AICAR to inhibit ER stress responses in an AMPK-independent manner. This may add to the potency of AICAR to prevent SFA-induced inflammation as compared to other AMPK activators, since ER stress is a key factor in metabolic overload-triggered inflammatory responses. Remarkably, other AMPK activators are either less potent or completely unable (A769662) to inhibit palmitate-induced inflammation. Although cumulative evidence from mouse genetic models of AMPK deficiency strongly supports anti-inflammatory roles of AMPK, it is still unclear, whether AMPK is similarly active in the human vs. mouse innate immune system. Furthermore, the phenotype of genetic AMPK deficiency is definitely influenced by compensatory responses to AMPK loss, whereas pharmacological AMPK activators are supplied to unperturbed cellular systems, making a comparison of the responses in these two settings difficult. Regardless of their impact on AMPK, several AMPK activators are known for AMPK-independent anti-inflammatory activities. This comprises mitochondrial complex I inhibition by biguanidines^21^, or targeting the NFκB activation pathway by salicylate^30^. Although AICAR was known to inhibit inflammation in an AMPK-independent manner^22^,^23^, molecular mechanisms remained unclear. The novelty of our data implies that under conditions of metabolic overload, inhibition of ER stress responses may contribute to the spectrum of anti-inflammatory AICAR effects. It is likely that the ability of AICAR to interfere with other aspects of pro-inflammatory macrophage signalling, as reported by others^19^,^22^,^23^, generates multi-pronged effects, resulting in the profound inhibition of inflammatory cytokine expression as observed in Fig. 1C.

Interestingly, AICAR does not attenuate initiation of ER stress responses, but inhibits mRNA and protein expression of all major transcriptional ER stress effectors, i.e. ATF4, spliced XBP1, and ATF6. Although initial results indicated a more selective response to the IRE1 branch of the ER stress, detailed examination of the IRE1 cellular and *in vitro* activities revealed that AICAR may act as a type I kinase inhibitor of IRE1^28^, inhibiting IRE1 autophosphorylation and at the same time activating its RNase activity. The downstream signalling of IRE1 is still blocked by AICAR, since it apparently inhibits expression of total mRNA, and as a consequence, spliced XBP1. Although splicing activity of IRE1 is not inhibited by AICAR, its effect on IRE1 kinase activity may affect IRE1 interaction with JNK^15^ and thus, contributes to attenuated JNK activity and subsequent inflammatory responses in AICAR-treated cells.

How AICAR inhibits ER stress-induced expression of ATF4, XBP1, and ATF6 remains unclear. To explore detailed mechanisms is complicated by fact that ER stress transcriptional effectors induce each other^31^,^32^. Furthermore, it is likely that AICAR also attenuates translation of ER stress effectors, as we observed that AICAR-treated macrophages contained reduced amounts of polysome-associated RNA (data not shown). Although AICAR may affect translation through AMPK-mediated inhibition of the mTORC1 pathway and phosphorylation of elongation factor 2^33^, persistence of AICAR inhibitory effects in the absence of its conversion to ZMP, despite blocking mTORC1 activity and elongation factor 2 phosphorylation (Fig. 2E and data not shown), suggests that other mechanisms of translational attenuation may operate. This is similar to our observations on AICAR-mediated inhibition of the peroxisome proliferator activated receptor γ translation in the absence of its conversion to ZMP^34^. Interestingly, mTORC1 inhibition induced either by rapamycin or AMPK activators is inferior to AICAR in alleviating palmitate-induced ER stress responses, arguing that the potency of AICAR is likely to represent a combination of transcriptional and translational effects.

Our data also suggest that cellular levels of the CNT and ENT transporters as well as adenosine kinase activity critically determine the outcome of the AICAR effect. Corroborating previous findings that modulating CNT and ENT expression influences intracellular ZMP accumulation and hence the magnitude of ZMP-mediated intracellular responses^26^, our study is the first to show that the endogenous CNT3 expression controls AICAR actions in macrophages. Of note, ENTs and CNTs are important drug targets to modulate the responses to nucleoside-based chemotherapies^35^. Similarly, we suggest that strategies aimed at enhancing ENT1 and CNT3 expression may promote AICAR anti-inflammatory and anti-ER-stress effects. Second, adenosine kinase activity may also determine the outcome of AICAR effects, switching it from ZMP-dependent (predominantly AMPK-mediated) to ZMP-independent pathways. Remarkably, in some cell contexts, such as acute myeloid leukemia, AMPK activation actually promotes ER stress^36^. Thus, it is interesting to test how adenosine kinase inhibitors will interact with AICAR systemically in the context of inflammatory diseases. We suggest that this combination can be more effective to suppress ER stress and inflammation and ameliorate metabolic disease. Considering that combinations with adenosine kinase inhibitor may allow to lower the range of AICAR concentrations inhibiting ER stress towards levels observed in plasma of AICAR-treated volunteers^37^, our mechanistic finding may be thus translatable into human clinical setting.

## Methods

### Cell isolation and culture

Human peripheral blood mononuclear cells were isolated from buffy coats of anonymous donors (DRK-Blutspendedienst Baden-Württemberg-Hessen, Institut für Transfusionsmedizin und Immunhämatologie, Frankfurt, Germany) using Ficoll density centrifugation. Following 1 h culture in serum-free RPMI medium, non-adherent cells were removed by vigorous washing. Remaining adhered monocytes were differentiated into macrophages by 7 d culture in RPMI 1640 medium containing 3% AB-positive human serum. Cells were treated (where indicated) with the following reagents: 500 μM AICAR, 50 μM etomoxir (both EMD Biosciences), 250 μM A769662, 0.5 μM ABT-702 (both Tocris), 1 μM R419 (Rigel Phramaceuticals), 100 nM thapsigargin (Enzo Life Sciences), 10 μM Ex527 (Selleckchem), 1 μg/ml tunicamycin, 3 mM sodium salicylate, 100 μM phenformin, 100 nM rapamycin or 20 μM dipyridamole (all Sigma-Aldrich). Palmitate (P-5585, Sigma-Aldrich) was prepared by diluting a 100 mmol/L stock solution in 70% ethanol/0.1 mol/L NaOH into 10% fatty acid-free low endotoxin BSA (A-8806, Sigma-Aldrich, adjusted to pH 7.4) to obtain a palmitate:BSA molar ratio of 6:1 and a final palmitate concentration 500 μM. Hypoxic incubations were performed using a hypoxic workstation with 1% O_2_, 94% N_2_, 5% CO_2_ at 37 °C (*Invivo*2 400, Ruskinn Technology). HEK293T cells were cultured in DMEM medium with 10% fetal calf serum, 100 U/ml penicillin, 100 μg/ml streptomycin.

Studies conform to the principles outlined in the Declaration of Helsinki and were approved by the ethics committee of the Faculty of Medicine at Goethe-University Frankfurt. The ethics committee waived the necessity of written informed consent when using the buffy coats from anonymized blood donors.

### siRNA transfection of macrophages

Silencing of AMPKα1 and SLC28A3 in human primary macrophages was achieved using corresponding siGENOME SMARTpools (Thermo Fisher Scientific) at 50 nM and Hiperfect transfection reagent (Qiagen). Cells were treated 72 h post-transfection.

### Quantitative PCR

Total RNA of primary human macrophages was isolated using PeqGold RNAPure kit (PeqLab) and transcribed using cDNA Synthesis kit (Fermentas). Quantitative PCR was performed with iQ SYBR green Supermix (Bio-Rad) using the CFX96 system from Bio-Rad. Primer sequences for quantitative PCR can be obtained upon request. Expression was normalized to β-microglobulin.

### XBP1 splicing analysis

To analyse XBP1 splicing 1 μg of RNA was reverse-transcribed. The cDNA was PCR amplified using primers designed to encompass the splicing site^38^ with DNA Taq polymerase (26 cycles at 94 °C 30 s, 60 °C 30 s, 72 °C 30 s). PCR products were resolved on 2% agarose gel, stained with ethidium bromide, and visualized using GenoSmart2 gel documentation system (VWR International).

### Western blot

Protein lysates were separated on polyacrylamide gels followed by transfer onto nitrocellulose membranes. Membranes were incubated with antibodies against phospho-ACC (Ser79) (#3661), phospho-AMPK (Thr172) (#2531), phospho-eIF2α (Ser51) (#3398), phospho-cJun (Ser73) (#3270), phospho-S6 ribosomal protein (Ser240/244) (#2215), ACC (#3662), AMPK (#2532) (Cell Signalling Technology), sXBP1 (6196, BioLegend), phospho-IRE1 (Ser724) (ab124945, Abcam), ATF6 (NBP1-75478, Novus Biologicals), ATF4 (sc-200), or nucleolin (sc-13057) (Santa Cruz Biotechnology) followed by IRDye 800-coupled secondary antibodies (LICOR Biosciences). Blots were visualized and quantified using the Odyssey imaging system (LICOR Biosciences).

### IL-1β detection in supernatants

Macrophage supernatants were analysed for IL-1β using a Cytometric Bead Array (CBA) (BD Biosciences) according to the manufacturer’s recommendations. Samples were measured using a LSRII/Fortessa flow cytometer (BD Biosciences) and analysed by FCAP Array software V1.0 (Soft Flow).

### HEK293T cell transfection

100000 cells plated in 6-well plates were cultured overnight and transfected with 1 μg plasmid coding for GFP (pmaxGFP, Lonza) or SLC28A3 (RC210847, Origene) using JetPRIME transfection reagent (Polyplus-transfection) according to manufacturer’s instructions.

### Triglyceride determination

Cell pellets were lysed in PBS containing 1% Triton X-100. Determination of triglycerides in lysates was carried out using a commercial kit from Roche Diagnostics according to manufacturer instructions.

### Lanthascreen analysis of AICAR binding to IRE1α

AICAR binding to recombinant baculovirus-expressed His-tagged dephosphorylated IRE1α purified from *Sf9* cells^39^ was assessed using Lanthascreen time-resolved fluorescence energy transfer assay (Thermo Scientific). A master mix (7.5 μL) consisting of dephosphorylated IRE1α (50 nM), 2 nM biotinylated anti-His-tag antibody (PV6089, Thermo Scientific), 2 nM streptavidin-europium (PV5899, Thermo Scientific) diluted in assay buffer PV3189 (Thermo Scientific), 1 mM DTT (D0632, Sigma-Aldrich) was incubated with 7.5 μL of 75 nM tracer (tracer 236, Thermo Scientific) in assay buffer for 60 min at room temperature. During the incubation time the plate (384 white low volume plate, 3674, Corning) was covered with foil. The assay was then read on an EnVision™ 2103 multi label microplate reader.

### DELFIA autophosphorylation assay

DELFIA autophosphorylation assay on recombinant dephosphorylated IRE1α was performed as described previously^39^.

### IRE1α RNase activity analysis

Ribonuclease activity of recombinant IRE1α was analysed using FRET derepression assay^40^. 22.5 nL of the RNA tracer (300 nM, Integrated DNA Technologies, sequence 5′6-FAM-CUG-AGU-CCG-CAG-CAC-UCA-G-3IABkFQ_1-3′) was added to the plate (384 black low volume plates, 3676, Corning) via the Echo ^®^ 550 acoustic dispenser (Labcyte). This was followed by 5 μL assay buffer (20 mM Hepes pH 7.5, 0.05% Triton X 100 v/v, 1 mM DTT). Then 5 μL of phosphorylated IRE1 (1 μg/mL) was added to the plate. The plate was centrifuged for 1 min at 1000 rpm. The plate was then read on an EnVision™ 2103 multi label microplate after 10 min.

### Statistical analysis

Data are presented as means ± SEM of at least three independent experiments. Data were analysed by one-way ANOVA with Bonferroni post hoc means comparison using Prism software (GraphPad). Differences were considered statistically significant for P < 0.05.

## Additional Information

**How to cite this article**: Boß, M. *et al*. AMPK-independent inhibition of human macrophage ER stress response by AICAR. *Sci. Rep*. **6**, 32111; doi: 10.1038/srep32111 (2016).

## Supplementary Material

Supplementary Information
