# Supplementary material for: AMPK-independent inhibition of human macrophage ER stress response by AICAR
Source: Sci Rep. 2016 Aug 26;6:32111. doi: 10.1038/srep32111 (PMC4999824; doi:10.1038/srep32111)
Supplement: Supplementary Information [file srep32111-s1.pdf]

Marcel Boß, Yvette Newbatt, Sahil Gupta, Ian Collins, Bernhard Brüne, and Dmitry Namgaladze

Figure S1

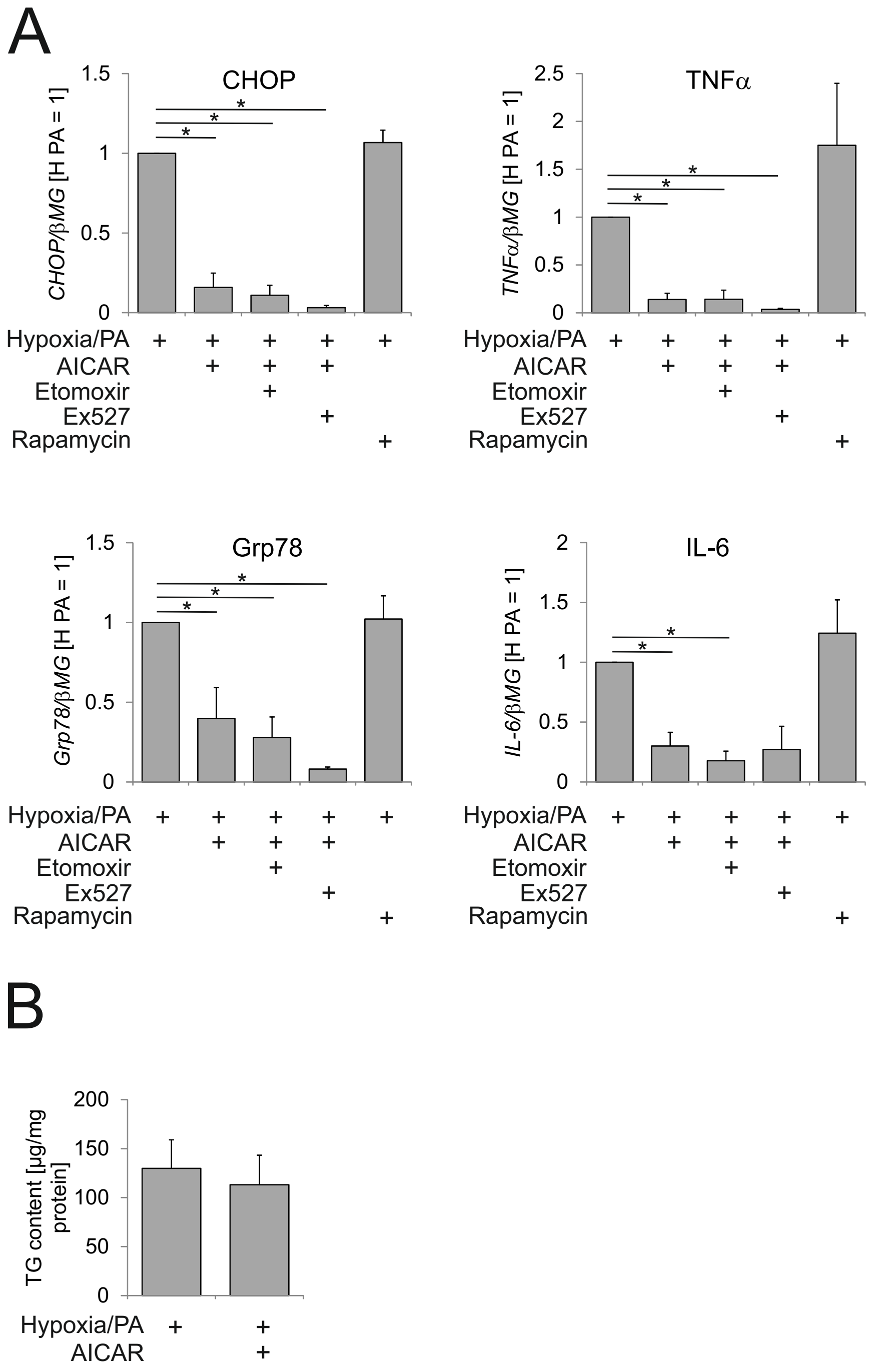

Supplementary Figure S1

(A) mRNA expression of genes in hypoxic macrophages treated for 24 h with palmitate in the presence of the indicated compounds. (B)Triglyceride content of hypoxic macrophages treated for 24 h with palmitate and AICAR. *, p<0.05.

AMPK-independent inhibition of human macrophage ER stress response by AICAR.

Marcel Boß, Yvette Newbatt, Sahil Gupta, Ian Collins, Bernhard Brüne, and Dmitry Namgaladze

Figure S2

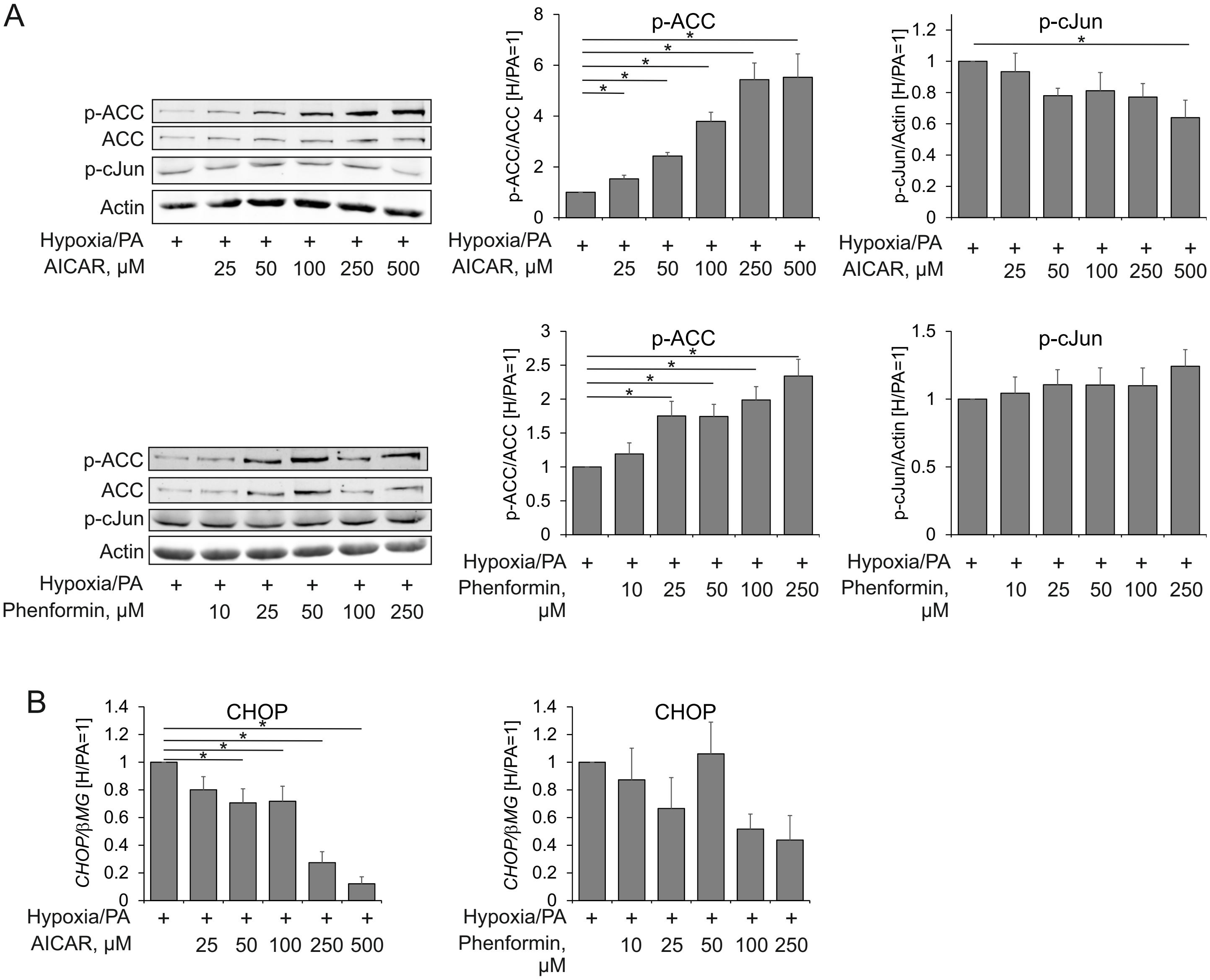

Supplementary Figure S2

(A) Western analysis and (B) mRNA expression of CHOP in hypoxic macrophages treated for 24 h with palmitate in the presence of the indicated concentrations of AICAR and phenformin. *, $p<0.05$.
